# NudCL2 is an autophagy receptor that mediates selective autophagic degradation of CP110 at mother centrioles to promote ciliogenesis

**DOI:** 10.1038/s41422-021-00560-3

**Published:** 2021-09-03

**Authors:** Min Liu, Wen Zhang, Min Li, Jiaxing Feng, Wenjun Kuang, Xiying Chen, Feng Yang, Qiang Sun, Zhangqi Xu, Jianfeng Hua, Chunxia Yang, Wei Liu, Qiang Shu, Yuehong Yang, Tianhua Zhou, Shanshan Xie

**Affiliations:** 1 The Children’s Hospital, Zhejiang University School of Medicine, National Clinical Research Center for Child Health, Hangzhou, Zhejiang China; 2 Department of Cell Biology, Zhejiang University School of Medicine, Hangzhou, Zhejiang China; 3 Institute of Gastroenterology, Sir Run Run Shaw Hospital, Zhejiang University School of Medicine, Hangzhou, Zhejiang China; 4 Cancer Center, Zhejiang University, Hangzhou, Zhejiang China; 5 Department of Molecular Genetics, University of Toronto, Toronto, ON Canada

**Keywords:** Cilia, Centrosome

## Abstract

Primary cilia extending from mother centrioles are essential for vertebrate development and homeostasis maintenance. Centriolar coiled-coil protein 110 (CP110) has been reported to suppress ciliogenesis initiation by capping the distal ends of mother centrioles. However, the mechanism underlying the specific degradation of mother centriole-capping CP110 to promote cilia initiation remains unknown. Here, we find that autophagy is crucial for CP110 degradation at mother centrioles after serum starvation in MEF cells. We further identify NudC-like protein 2 (NudCL2) as a novel selective autophagy receptor at mother centrioles, which contains an LC3-interacting region (LIR) motif mediating the association of CP110 and the autophagosome marker LC3. Knockout of *NudCL2* induces defects in the removal of CP110 from mother centrioles and ciliogenesis, which are rescued by wild-type NudCL2 but not its LIR motif mutant. Knockdown of CP110 significantly attenuates ciliogenesis defects in *NudCL2*-deficient cells. In addition, *NudCL2* morphants exhibit ciliation-related phenotypes in zebrafish, which are reversed by wild-type NudCL2, but not its LIR motif mutant. Importantly, *CP110* depletion significantly reverses these ciliary phenotypes in *NudCL2* morphants. Taken together, our data suggest that NudCL2 functions as an autophagy receptor mediating the selective degradation of mother centriole-capping CP110 to promote ciliogenesis, which is indispensable for embryo development in vertebrates.

## Introduction

Primary cilia are microtubule-based organelles that project from the surface of vertebrate cells to transduce extracellular signals into intracellular responses,^1,2^ and are of crucial importance in vertebrate development and homeostasis maintenance.^3–7^ Defects in cilia function are involved in multiple human syndromes that are collectively called ciliopathies, including situs inversus, congenital heart defects, cystic kidney disease and so on.^8–12^ Primary cilia form in the interphase, and are resorbed prior to mitosis.^13–15^ In G0 or early G1 phase, mother centrioles convert into basal bodies, dock to the plasma membrane through their distal appendages, and nucleate ciliary axonemes to form primary cilia.^16^ Although ciliogenesis regulation has been intensively studied, the mechanism of ciliogenesis initiation remains unclear.

Centriolar coiled-coil protein 110 (CP110), originally characterized as a cyclin-dependent kinase substrate, caps the distal ends of both mother and daughter centrioles, and is essential for centriole duplication and ciliogenesis initiation in mammalian cells.^7,17–23^ The removal of CP110 from mother centrioles is prerequisite for enabling axoneme outgrowth and appears to be one of the earliest steps in initiating ciliogenesis.^7,19–23^ CP110 antagonizes the action of centrosomal protein of 290 kDa (CEP290) to suppress the recruitment of small GTPase Rab8a, resulting in the inhibition of cilia formation.^21^ However, the mechanism by which mother centriole-localized CP110 is degraded to promote ciliogenesis remains unknown.

Autophagy is a lysosome-dependent program that degrades cytoplasmic proteins or organelles to maintain cellular homeostasis in response to different types of stresses.^24,25^ Previous reports have shown that autophagy is involved in the regulation of ciliogenesis by degrading ciliary proteins such as satellite oral-facial-digital syndrome 1 (OFD1) and intraflagellar transport 20 (IFT20).^26–28^ Nevertheless, it is unknown whether autophagy plays a role in CP110 degradation to initiate ciliogenesis.

In this manuscript, we find that autophagy is crucial for the specific removal of CP110 from mother centrioles and ciliogenesis. Further data show that after serum starvation, an Hsp90 co-chaperone NudC-like protein 2 (NudCL2) functions as a previously uncharacterized autophagy receptor mediating the selective autophagic degradation of CP110 at mother centrioles, which is essential for ciliogenesis initiation and vertebrate development.

## Results

### Autophagic degradation of CP110 contributes to ciliogenesis

It is well known that serum starvation can physiologically trigger ciliogenesis in many types of cultured cells.^29,30^ A number of studies have shown that CP110 removal from mother centrioles is required for ciliogenesis initiation after serum starvation.^7,19–23^ Here, we verified that serum deprivation induced CP110 removal from mother centrioles, promoted ciliogenesis, and decreased the protein level of CP110 in mouse embryonic fibroblast (MEF) cells (Supplementary information, Fig. S1). There are two major degradation pathways for protein turnover: the ubiquitin-proteasome pathway and autophagy-lysosome pathway.^31^ Thus, we determined which pathway is responsible for CP110 degradation at mother centrioles. Since proteins fated for ubiquitin-proteasomal degradation are usually conjugated to ubiquitin, we examined whether CP110 is ubiquitylated in cultured MEF cells at different time points after serum starvation. Given that centrosomal protein 97 (CEP97) has been reported to be degraded via the ubiquitin-proteasome system to facilitate ciliation, we chose CEP97 as a positive control.^32^ Our results revealed that the ubiquitination level of CP110 did not obviously change, whereas the ubiquitination level of CEP97 was dramatically increased during ciliogenesis initiation induced by serum starvation (Supplementary information, Fig. S2), suggesting that the ubiquitin-proteasome pathway may not be directly involved in CP110 degradation during ciliogenesis.

**Fig. 1:**
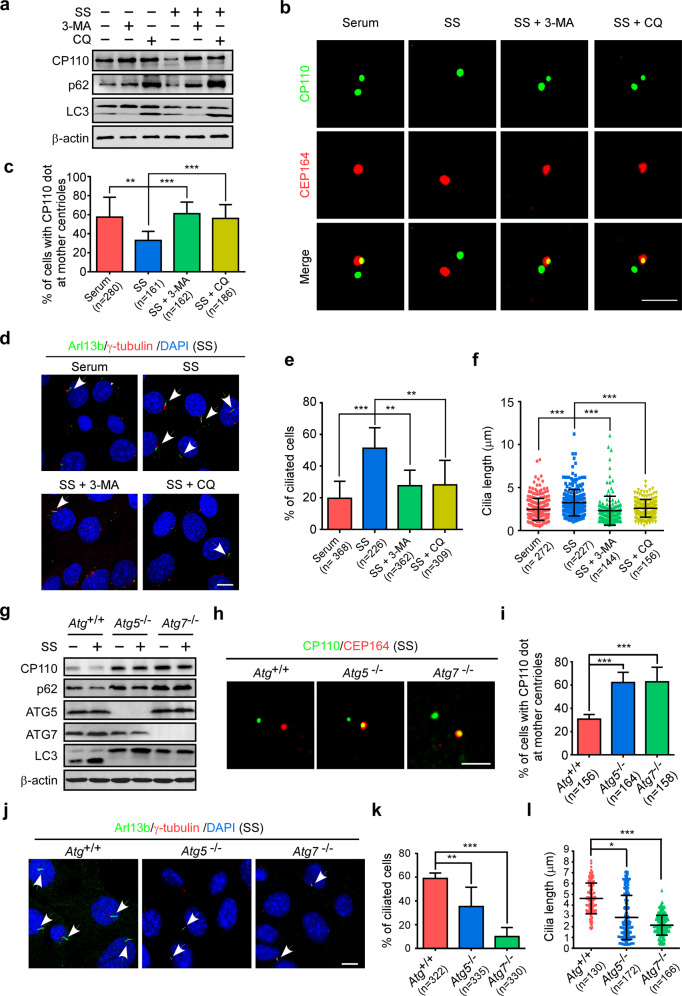
Autophagic degradation of CP110 contributes to ciliogenesis. **a**–**f** MEF cells with or without serum starvation (SS) were treated with 5 mM 3-MA, or 20 μM CQ for 24 h, and applied for the following analyses. Western analysis showed the protein levels of endogenous CP110, LC3 and p62 (**a**). β-actin was used as a loading control. Immunofluorescence analyses were carried out by using anti-CEP164 and anti-CP110 antibodies (**b**). CEP164, a marker of mother centrioles. Scale bar, 2 µm. The percentage of cells with CP110 dot at mother centrioles was calculated (**c**). Immunostaining of γ-tubulin and ADP ribosylation factor like GTPase 13b (Arl13b) in the indicated cells is shown (**d**). Arl13b and γ-tubulin are cilia and centrosome markers, respectively. The arrowheads indicate cilia. Scale bar, 10 µm. The cells with cilia were counted (**e**). Cilia length was also measured using ImageJ software (**f**). **g**–**l** Autophagy-deficient *Atg5*^*–/–*^ or *Atg7*^*–/–*^ MEF cells cultured with or without serum were subjected to the following analyses. Western blotting showed the levels of the indicated proteins (**g**). Immunofluorescence analyses were carried out by using anti-CEP164 and anti-CP110 antibodies (**h**). Scale bar, 2 µm. The percentage of cells with CP110 dot at mother centrioles was counted (**i**). Immunostaining of γ-tubulin and Arl13b in the indicated cells is shown (**j**). The arrowheads indicate cilia. Scale bar, 10 µm. The ciliated cells were calculated (**k**) and cilia length was determined using ImageJ software (**l**). Quantitative data are expressed as the means ± SD (at least three independent experiments). *n*, sample size. *P < 0.05, **P < 0.01, and ***P < 0.001; ns, not significant (*P* > 0.05); Student’s *t*-test.

To explore the role of autophagy in CP110 degradation and ciliogenesis, we employed the autophagy inhibitors 3-methyladenine (3-MA) and chloroquine (CQ) and autophagy-deficient cells to block autophagy pathway. The results showed that 3-MA or CQ was able to block CP110 degradation and its elimination from mother centrioles, and suppress ciliogenesis during serum starvation (Fig. 1a–f). Further experiments in autophagy-deficient MEF cells also revealed that deletion of the essential autophagy gene *Atg5* or *Atg7* suppressed CP110 degradation, CP110 removal from mother centrioles, and ciliogenesis under serum deprivation (Fig. 1g–l). Taken together, these data suggest that autophagy promotes CP110 removal from mother centrioles to initiate ciliogenesis.

### CP110 is associated with the autophagosome marker LC3 at mother centrioles

**Fig. 2:**
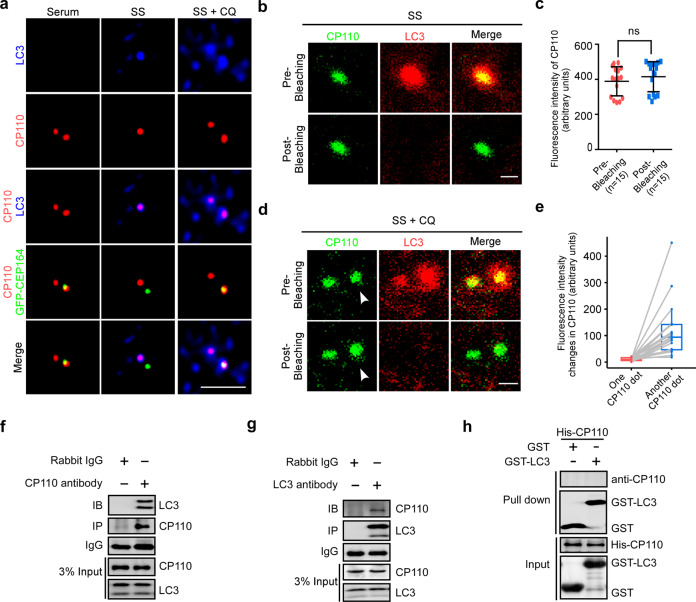
CP110 is associated with LC3 at mother centrioles. **a** MEF cells transfected with GFP-CEP164 were cultured with or without serum for 24 h, treated with or without 20 μM CQ, and processed for immunostaining analysis with anti-CP110 and anti-LC3 antibodies. Scale bar, 2 µm. **b**–**e** MEF cells were starved for 24 h, treated with or without 20 μM CQ, stained with anti-CP110 and anti-LC3 antibodies, and subjected to FRET analysis by acceptor photobleaching. The LC3 dots were photobleached. Representative images of FRET experiments are shown (**b**, **d**). The dots with FRET-positive signals are labeled by white arrowheads. Scale bar, 0.5 µm. CP110 fluorescence intensities at daughter centrioles before (pre-) and after (post-) photobleaching are shown in starved cells (**c**). The quantification of changes in the CP110 fluorescence intensity at centrioles of starved cells with CQ treatment after photobleaching is presented (**e**). **f**, **g** MEF cells cultured with serum were processed for co-IP analysis with anti-CP110 (**f**) or anti-LC3 (**g**) antibodies. **h** In vitro interaction of purified GST-LC3 with His-CP110. Quantitative data are expressed as the means ± SD (at least three independent experiments). *n*, sample size. ns, not significant (*P* > 0.05); Student’s *t*-test.

Given that LC3 has been reported to interact with organelles or proteins to target them for autophagic degradation,^33,34^ and that autophagy is essential for CP110 removal from mother centrioles, we hypothesized that LC3 may influence CP110 removal from mother centrioles. Our western blotting and immunostaining assays showed that LC3 depletion increased CP110 levels and impaired CP110 removal from mother centrioles in MEF cells under serum starvation (Supplementary information, Fig. S3). We then tested whether LC3 is associated with CP110 at mother centrioles, and detected the localization of LC3 with CP110, which decorates daughter centrioles under serum starvation.^7,19–23^ Our data revealed that few LC3 was detected at mother or daughter centrioles in most cells cultured with serum, while LC3 was clearly localized at only daughter centrioles with CP110 after serum deprivation (Fig. 2a). Inhibition of serum deprivation-induced autophagy with CQ led to the co-localization of LC3 with CP110 at both the mother and daughter centrioles (Fig. 2a). Moreover, fluorescence resonance energy transfer (FRET) assays displayed that there existed no significant change in CP110 fluorescence intensity at daughter centrioles after LC3 bleaching upon serum starvation (Fig. 2b, c), indicating that LC3 does not directly interact with CP110 at daughter centrioles. However, we found a FRET-negative signal of CP110 at one centriole and a FRET-positive signal of CP110 at another centriole in the majority of cells when starved cells were treated with CQ (Fig. 2d, e). Collectively, these data suggest that LC3 may be associated with CP110 at mother centrioles but not at daughter ones.

To confirm the association of LC3 and CP110, we performed co-immunoprecipitation (co-IP) experiments and discovered that endogenous CP110 was able to interact with LC3 in cells (Fig. 2f, g). Unexpectedly, the in vitro pull-down assay showed that there was no direct interaction between LC3 and CP110 (Fig. 2h). One possible interpretation is that there may exist a mediator to bridge the association between LC3 and CP110.

### NudCL2 mediates the interaction between LC3 and CP110

To determine the molecule that mediates the interaction between LC3 and CP110, we employed co-IP combined with mass spectrometry (IP-MS) to identify CP110- or LC3-interacting proteins (Supplementary information, Fig. S4a). CP110 and LC3 interactomes further overlapped with known centrosome/cilia-associated proteins,^35^ and 21 protein candidates were selected (Supplementary information, Fig. S4b). We reasoned that the depletion of the mediator would disrupt the LC3–CP110 interaction to inhibit the autophagic degradation of CP110, resulting in elevated CP110 levels under serum starvation. Thus, we knocked down each mediator candidate by using siRNAs to examine their effects on the protein stability of CP110 (Supplementary information, Fig. S4c, d). Western blot analysis showed that only NudCL2 knockdown obviously increased CP110 levels among these candidates.

**Fig. 3:**
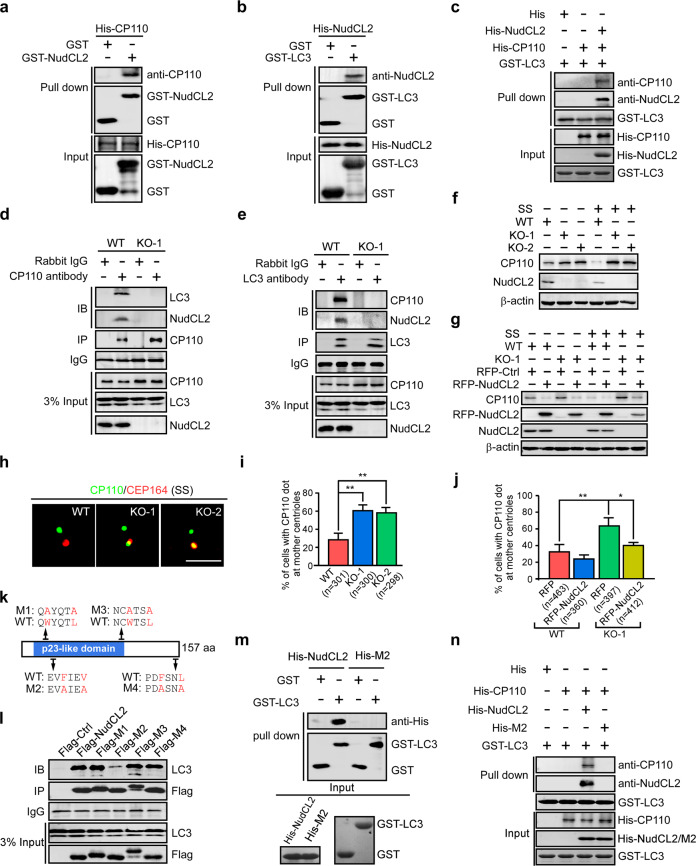
NudCL2 mediates the interaction between LC3 and CP110. **a** GST pull-down analyses of purified GST-NudCL2 with His-CP110. **b** GST pull-down analyses of purified GST-LC3 with His-NudCL2. **c** In vitro protein interaction of purified GST-LC3 with His-CP110 and His-NudCL2. **d**, **e** Wild-type (WT) and *NudCL2* knockout (KO-1) MEF cells were applied for co-IP analysis with anti-CP110 or anti-LC3 antibodies, respectively. 3% of total input is shown. **f**–**j** WT and *NudCL2* KO MEF cells were transfected with the indicated plasmids, cultured with or without serum for 24 h, and processed for the following analyses. Western blot analysis showed the expression of the indicated proteins (**f**, **g**). β-actin was used as a loading control. Immunofluorescence analyses were carried out by using anti-CEP164 and anti-CP110 antibodies (**h**). Scale bar, 2 µm. The percentage of cells with CP110 dots at mother centrioles was calculated (**i**, **j**). **k** Schematic diagram shows the possible LIR motifs (W/F/YxxL/I/V) of NudCL2. The point mutations (depicted in red) in four putative LIR motifs are defined as M1, M2, M3, and M4, respectively. **l** MEF cells transfected with the indicated plasmids were subjected to co-IP experiments. 3% of total input is shown. **m** GST pull-down analysis of purified GST-LC3 with His-NudCL2 or His-M2. **n** In vitro protein interaction of purified GST-LC3 with His-CP110 and His-NudCL2 or His-M2. Quantitative data are expressed as the means ± SD (at least three independent experiments). *n*, sample size. *P < 0.05 and **P < 0.01; Student’s *t*-test.

To verify the interaction among LC3, CP110 and NudCL2, we performed reciprocal co-IP experiments in MEF cells and discovered that endogenous LC3, CP110 and NudCL2 were able to interact with each other (Supplementary information, Fig. S4e–g). Moreover, GST pull-down assays confirmed that purified NudCL2 not only directly interacted with either LC3 or CP110 (Fig. 3a, b), but also bridged the interaction between LC3 and CP110 (Fig. 3c). To investigate the role of NudCL2 in mediating the LC3–CP110 interaction in cells, we generated *NudCL2* knockout (KO) MEF cell lines by using the CRISPR/Cas9 system. Two *NudCL2* KO cell lines (KO-1 and KO-2) were established and verified by Sanger sequencing and western blotting (Supplementary information, Fig. S5). Further co-IP analyses showed that the interaction between LC3 and CP110 was abolished in *NudCL2* KO cells (Fig. 3d, e), indicating that NudCL2 mediates the LC3–CP110 interaction in cells.

To delineate the region that mediates the interaction between NudCL2 and CP110. We divided NudCL2 into five fragments and found that the median and C-terminal regions of NudCL2 were able to interact with CP110 except for its N-terminal fragment (1–32 aa) (Supplementary information, Fig. S6a, b). Meanwhile, we also observed that only the median region of CP110 (49–639 aa) bound to NudCL2 (Supplementary information, Fig. S6c, d). These results suggest that NudCL2 and CP110 may have a multi-domain interaction pattern.

Then, we tried to confirm the role of NudCL2 in regulating the stability of CP110 in *NudCL2* KO cells. The data revealed that deletion of NudCL2 clearly suppressed CP110 degradation in serum-starved MEF cells (Fig. 3f), which was efficiently rescued by ectopic expression of NudCL2 (Fig. 3g). To determine whether NudCL2 is involved in CP110 degradation at mother centrioles, we assessed the effect of *NudCL2* knockout on CP110 degradation at mother centrioles. Our data displayed that deletion of NudCL2 significantly inhibited CP110 removal from mother centrioles in MEF cells cultured without serum (Fig. 3h, i), which was also reversed by exogenous expression of NudCL2 (Fig. 3j).

Since NudCL2 mediates LC3–CP110 interaction and is involved in CP110 removal at mother centrioles, we hypothesized that NudCL2 may function as an autophagy receptor to induce CP110 degradation at mother centrioles. To test this hypothesis, we carefully analyzed the amino acid sequence of NudCL2 and identified four potential LC3-interacting region (LIR) motifs (Fig. 3k). To define which LIR motif is required for the binding of NudCL2 to LC3, a mutation of each LIR motif was generated and subjected to co-IP assays (Fig. 3l). The results showed that only the ectopic expression of NudCL2 with the mutation in the second LIR motif (NudCL2-M2) robustly reduced the interaction between NudCL2 and LC3. GST pull-down assays also confirmed that NudCL2-M2 failed to interact with LC3 and mediate the interaction between LC3 and CP110 in vitro (Fig. 3m, n). Taken together, these data indicate that NudCL2 functions as an autophagy receptor mediating the interaction between LC3 and CP110.

### NudCL2 and CP110 are removed from mother centrioles during cilliogenesis

**Fig. 4:**
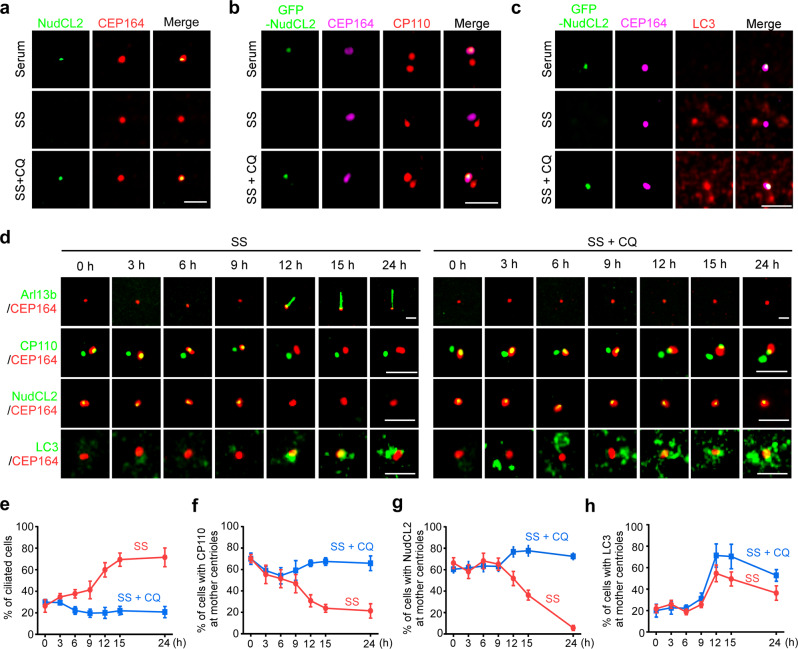
NudCL2 and CP110 are removed from mother centrioles during ciliogenesis. **a** MEF cells were cultured with or without serum starvation, treated with or without 20 μM CQ, and subjected to immunofluorescence analysis with anti-NudCL2 and anti-CEP164 antibodies. **b** MEF cells were transfected with GFP-NudCL2 and applied for immunofluorescence analysis with anti-CP110 and anti-CEP164 antibodies under the indicated treatments. **c** MEF cells transfected with GFP-NudCL2 were stained with anti-LC3 and anti-CEP164 antibodies under the indicated treatments. **d**–**h** MEF cells treated with or without 20 μM CQ were starved for different times, and then subjected to immunostaining analyses with the indicated antibodies. The percentages of ciliated cells (**e**), cells with CP110 dot at mother centrioles (**f**), cells with NudCL2 dot at mother centrioles (**g**), and cells with LC3-positive puncta at mother centrioles (**h**) are shown. Scale bars, 2 µm.

Given that autophagy promotes CP110 removal from mother centrioles to initiate ciliogenesis, and that NudCL2 is an autophagy receptor mediating the interaction between LC3 and CP110, we speculated that NudCL2 may localize at mother centrioles. Immunofluorescence analysis of NudCL2 and CEP164 revealed that NudCL2 mainly decorated mother centrioles in MEF cells with serum (Fig. 4a; Supplementary information, Fig. S7). Upon serum deprivation, NudCL2 obviously disappeared from mother centrioles, which was effectively blocked by CQ treatment, suggesting that NudCL2 may be degraded at mother centrioles via autophagy. To explore how NudCL2 is recruited to mother centrioles, we depleted several mother centriole-localized proteins,^23,36^ and found that knockdown of centrosomal protein 83 (CEP83), centrosomal protein 89 (CEP89) or Tau tubulin kinase 2 (TTBK2) significantly reduced the localization of NudCL2 at mother centrioles (Supplementary information, Fig. S8). Further co-IP assays revealed that NudCL2 interacted with CEP83, CEP89 or TTBK2. These data suggest that CEP83, CEP89 and TTBK2 may be involved in NudCL2 recruitment to mother centrioles.

To assess the association among NudCL2, CP110 and LC3 at mother centrioles, GFP-NudCL2-expressed cells were co-stained with CP110 or LC3 (Fig. 4b, c). The results showed that GFP-NudCL2 co-localized with CP110 at mother centrioles in cells cultured with serum. Serum starvation induced removal of GFP-NudCL2, CP110 and LC3 from mother centrioles, which was reversed by CQ treatment, implying that the elimination of both NudCL2 and CP110 from mother centrioles may be induced by autophagic degradation.

To investigate the association between the role of NudCL2 in CP110 degradation at mother centrioles and ciliogenesis, we observed the dynamic localization of CP110, NudCL2, and LC3 at mother centrioles after serum deprivation (Fig. 4d–h). At the beginning of serum starvation, the percentage of cells with cilia increased slowly, while both CP110 and NudCL2 were localized at mother centrioles with few LC3 signals in the majority of cells. From 9 h to 12 h post serum deprivation, LC3-positive puncta began to accumulate at mother centrioles, accompanied by the disappearance of NudCL2 and CP110 from mother centrioles, while the percentage of ciliated cells increased rapidly. More importantly, CQ treatment blocked the removal of NudCL2 and CP110 from mother centrioles and inhibited ciliogenesis, especially after 9 h of serum starvation.

### NudCL2 facilitates ciliogenesis by promoting CP110 removal at mother centrioles

**Fig. 5:**
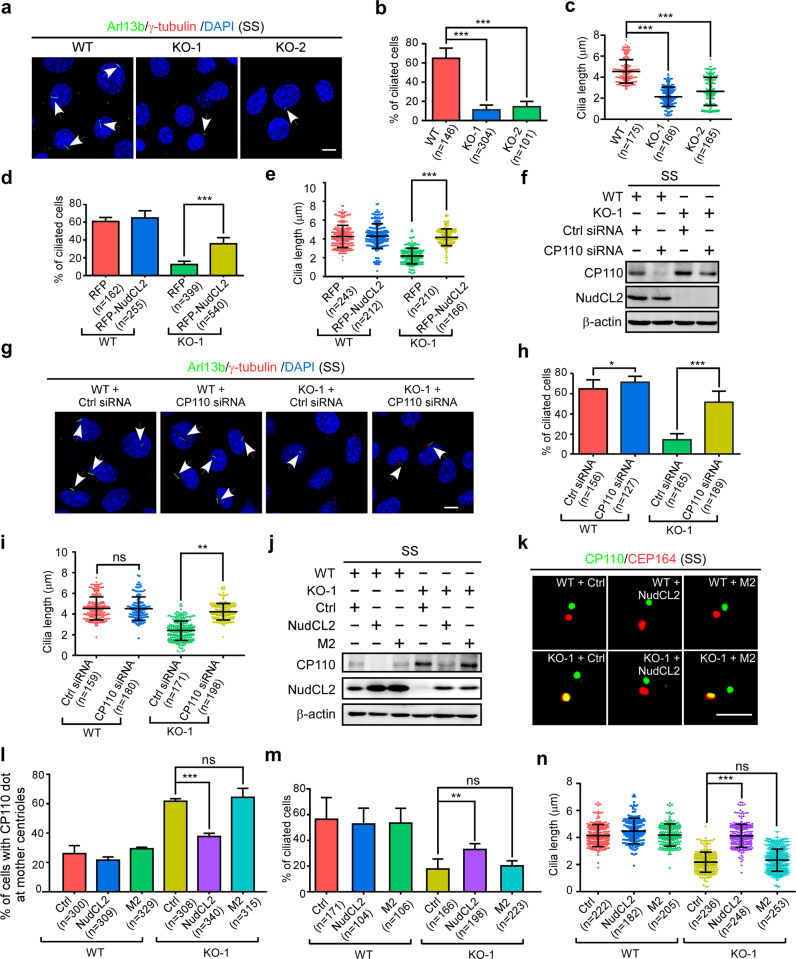
NudCL2 facilitates ciliogenesis by mediating CP110 degradation at mother centrioles. **a**–**c** WT and *NudCL2* KO MEF cells were starved for 24 h, and subjected to the following analyses. Immunostaining of cells with anti-Arl13b and anti-γ-tubulin antibodies is shown (**a**). Scale bar, 10 µm. DNA was visualized using DAPI. The proportion of cells with cilia was calculated (**b**), and cilia length was measured using ImageJ software (**c**). **d**, **e** WT and *NudCL2* KO MEF cells transfected with the indicated plasmids were cultured without serum for 24 h. The ciliated cells were counted (**d**). Cilia length was determined using ImageJ software (**e**). **f**–**i** WT and *NudCL2* KO MEF cells treated with the indicated siRNAs were starved for 24 h, and subjected to the following analyses. Western blotting showed the expression of CP110 and NudCL2 (**f**). β-actin, a loading control. Immunofluorescence analysis was carried out by using anti-Arl13b and anti-γ-tubulin antibodies (**g**). Cilia are indicated by white arrows. Scale bar, 10 µm. DNA was stained by DAPI. The ciliated cells were counted (**h**) and cilia length was measured using ImageJ software (**i**). **j**–**n** WT and *NudCL2* KO MEF cells infected with the indicated lentiviruses were cultured without serum for 24 h, and processed for the following analyses. Western blot analysis revealed NudCL2 and CP110 expression (**j**). Immunofluorescence analyses were carried out with anti-CEP164 and anti-CP110 antibodies (**k**). Scale bar, 2 µm. The percentage of cells with CP110 dots at mother centrioles was counted (**l**). The percentage of cells with cilia was calculated (**m**). Cilia length was also determined using ImageJ software (**n**). Quantitative data are expressed as the means ± SD (at least three independent experiments). *n*, sample size. *P < 0.05, **P < 0.01, and ***P < 0.001; ns, not significant (*P* > 0.05); Student’s *t*-test.

Since specific removal of CP110 at mother centriole is crucial for ciliogenesis initiation,^7,19–23^ and NudCL2 facilitates CP110 degradation at mother centrioles, we examined whether NudCL2 plays a role in ciliation. Our results showed that deletion of NudCL2 led to a robust reduction in ciliogenesis in MEF cells upon serum starvation (Fig. 5a–c), which was significantly reversed by the ectopic expression of NudCL2 (Fig. 5d, e). Then, we investigated if NudCL2 promotes ciliogenesis by degrading CP110. Our data revealed that knockdown of CP110 significantly rescued the defects of ciliogenesis in either *NudCL2* knockout or depleted cells (Fig. 5f–i; Supplementary information, Fig. S9). Additionally, we examined whether NudCL2 affects the ciliogenesis events earlier than CP110 removal,^23,36–40^ and found that NudCL2 deletion did not significantly influence distal appendage assembly, vesicle docking or TTBK2 recruitment (Supplementary information, Fig. S10). These data indicate that NudCL2 may mediate CP110 degradation to promote ciliogenesis.

Given that the LIR motif of NudCL2 is required for its binding to LC3, we reasoned that the LIR motif may contribute to CP110 degradation and subsequently promote ciliogenesis. We stably expressed wild-type NudCL2 or NudCL2-M2 in *NudCL2* KO MEF cells. The results showed that *NudCL2* deletion blocked CP110 degradation under serum starvation, which was reversed by the ectopic expression of wild-type NudCL2 but not NudCL2-M2 (Fig. 5j). Immunofluorescence localization of CP110 and CEP164 confirmed that ectopic expression of wild-type NudCL2 but not NudCL2-M2 facilitated the removal of mother centriole-localized CP110 in *NudCL2* KO MEF cells cultured without serum (Fig. 5k, l). Moreover, wild-type NudCL2 but not NudCL2-M2 was able to significantly rescue the defects of ciliogenesis in *NudCL2* KO MEF cells (Fig. 5m, n). Thus, these data suggest that NudCL2 plays crucial roles in ciliogenesis via CP110 degradation at mother centrioles.

### NudCL2 is required for ciliogenesis during zebrafish development

**Fig. 6:**
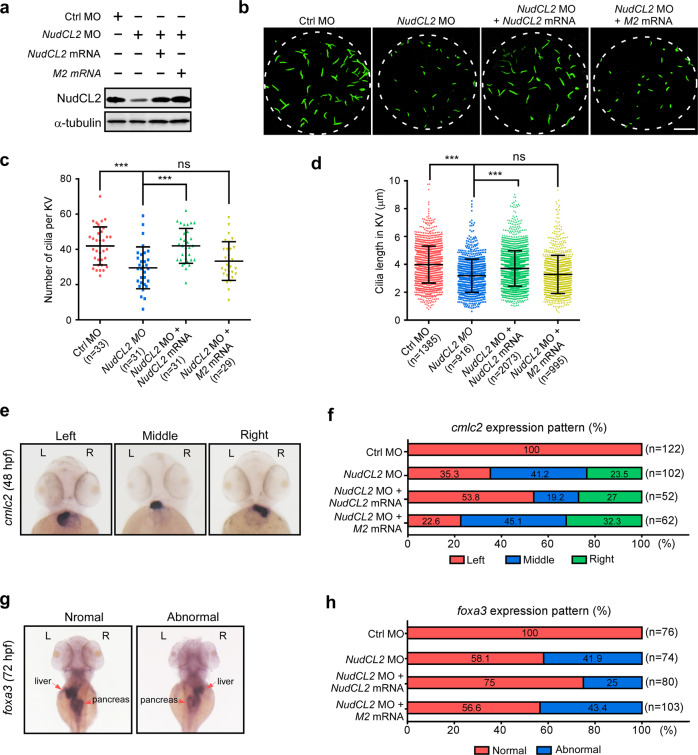
NudCL2 is essential for ciliogenesis during zebrafish development. Embryos were injected with the indicated MOs and mRNAs, and collected at different times. **a** Western blot analysis of NudCL2 expression. α-tubulin was used as a loading control. **b** Immunofluorescence of cilia in KVs with anti-acetylated-α-tubulin antibody (green) at 6–8 somite stages. Scale bar, 10 µm. **c**, **d** Cilia number per KV was calculated and cilia length was measured using ImageJ software. **e**–**h** Whole-mount in situ hybridization with *cmlc2* probe at 48 hpf or *foxa3* probe at 72 hpf. Liver and pancreas are indicated by arrows and arrowheads, respectively. Quantification of the expression patterns of *cmlc2* or *foxa3* is shown (**f**, **h**). The data are presented as means ± SD derived from at least three independent experiments. *n*, sample size. ***P < 0.001; ns, not significant (*P* > 0.05); Student’s *t*-test.

To determine whether NudCL2 is an important player in ciliogenesis during vertebrate development, we cloned zebrafish *NudCL2* (*Nudcd2*, NM_001003539.1; GenBank) and found that it encodes a deduced 157-aa protein with a conserved p23-like domain (Supplementary information, Fig. S11a).^41–44^ Zebrafish NudCL2 shared high homology with human NudCL2 (identity, 70.1%; similarity, 81.5%) (Supplementary information, Fig. S11b). Then, we designed morpholino antisense oligonucleotides (MOs) to block *NudCL2* mRNA translation. Our data revealed that the *NudCL2* MO injection obviously decreased the protein level of endogenous NudCL2 in zebrafish embryos (Fig. 6a). *NudCL2* morphants exhibited pericardial edema and curved bodies at 72 h post fertilization (hpf), which were significantly reversed by exogenous expression of *NudCL2* mRNA but not *NudCL2-M2* mRNA (Supplementary information, Fig. S12).

Since Kupffer′s vesicle (KV) is a ciliated organ formed during embryogenesis in zebrafish,^45^ we tested whether NudCL2 influences ciliation in KVs. The whole-mount immunofluorescence data showed that *NudCL2* knockdown significantly decreased cilia number and length in KVs, which was significantly rescued by ectopic expression of *NudCL2* mRNA but not *NudCL2-M2* mRNA (Fig. 6b–d). Given that KV is essential for governing the left–right asymmetry of zebrafish embryo,^46^ we examined the position of cardiac primordia (*cmlc2*, cardiac myosin light chain 2) and the expression pattern of the primordial liver and pancreas marker (*foxa3*, forkhead box protein a3) during zebrafish embryogenesis. Whole-mount in situ hybridization revealed that the control morphants exhibited normal expression pattern of *cmlc2* and *foxa3* (the liver on the left side and the pancreas on the right side) in visceral organs (Fig. 6e–h). *NudCL2* morphants displayed abnormal expression pattern of *cmlc2* and *foxa3*, which was also significantly reversed by the ectopic expression of *NudCL2* mRNA but not *NudCL2-M2* mRNA, implying an indispensable function of NudCL2 in the left–right asymmetry determination. Taken together, these data suggest that NudCL2 plays a critical role in ciliogenesis and cilia-mediated developmental processes via its LIR motif.

### CP110 mediates the role of NudCL2 in ciliogenesis during embryogenesis

**Fig. 7:**
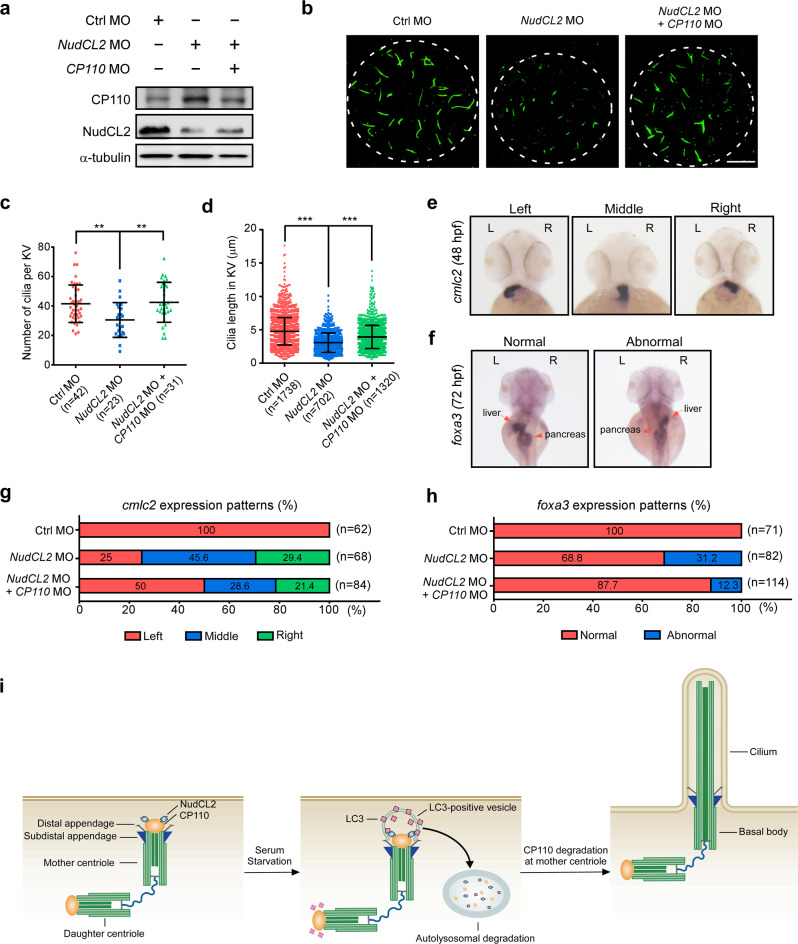
Knockdown of *CP110* reverses the ciliary defects in *NudCL2* morphants. Embryos were injected with the indicated MOs and harvested at different times. **a** Immunoblot analysis of NudCL2 and CP110 expression. α-tubulin, a loading control. **b**–**d** Immunofluorescence of cilia with anti-acetylated-α-tubulin antibody in KVs at 6–8 somite stages (**b**). Scale bar, 10 µm. The cilia number per KV was calculated (**c**). Cilia length in KV was measured using ImageJ software (**d**). **e**–**h** Whole-mount in situ hybridization with *cmlc2* probe (48 hpf) and *foxa3* probe (72 hpf). Liver and pancreas are indicated by arrows and arrowheads, respectively. The percentage of the expression patterns of *cmlc2* and *foxa3* was calculated (**g**, **h**). The data are presented as means ± SD derived from at least three independent experiments. *n*, sample size. **P < 0.01 and ***P < 0.001 Student’s *t*-test. **i** Working model of NudCL2-mediated autophagic degradation of CP110 at mother centrioles to initiate ciliogenesis. See text for details.

Based on the data that NudCL2 enhances ciliogenesis by promoting CP110 degradation at mother centrioles in mammalian cells, we explored whether NudCL2 regulates cilia-associated developmental processes via CP110 in zebrafish. We performed a series of rescue experiments by depleting *CP110* in *NudCL2* morphants. Western blotting showed that the protein level of CP110 was increased in *NudCL2* morphants, which was reversed by co-injection with *CP110* MO (Fig. 7a). Knockdown of *CP110* in *NudCL2* morphants significantly rescued the ciliary phenotypes, including pericardial edema and curved bodies (Supplementary information, Fig. S13). Whole-mount immunofluorescence showed that cilia defects in the KV of *NudCL2* morphants were significantly reversed by *CP110* depletion (Fig. 7b–d). Furthermore, depletion of *CP110* effectively reversed the left–right asymmetry defects induced by *NudCL2* knockdown (Fig. 7e–h). Taken together, these data suggest that NudCL2 regulates the cilia-associated developmental events via CP110 in zebrafish.

## Discussion

In this study, we provide evidence that NudCL2 serves as a hitherto unrecognized autophagy receptor mediating CP110 removal from mother centrioles, which is crucial for ciliogenesis initiation (Fig. 7i). In most cells cultured with serum, CP110 caps the distal ends of both mother and daughter centrioles, whereas NudCL2 preferentially localizes at mother centrioles. Upon serum deprivation, LC3 begins to interact with NudCL2 at mother centrioles to induce autophagic degradation of CP110, resulting in ciliogenesis.

The level and localization of CP110 at centrosomes are tightly regulated in a cell cycle-dependent manner.^17^ CP110 levels are low in G0 or early G1 phase, but markedly increase as cells progress through the G1/S transition, and then start to decline in G2 and M phases.^17^ It has been reported that the ubiquitin-proteasome system regulates CP110 degradation to control centriole duplication during G2 phase.^47^ In G0 phase, CP110 is removed from mother centrioles to initiate ciliogenesis;^7,19–23^ however, the underlying molecular mechanism is largely unexplored. Recent studies have identified CEP97 as an important regulator stabilizing CP110 at mother centrioles to inhibit ciliogenesis.^19^ Upon serum withdrawal, TTBK2 is recruited to basal body to phosphorylate M-Phase Phosphoprotein 9 (MPP9), which induces the de-localization of the CP110–CEP97 complex from mother centrioles to enhance cilia formation.^48,49^ Loss of kinesin family member 24 (Kif24) also results in CP110 de-localization and leads to unscheduled ciliogenesis.^50^ Here, our data clearly show that autophagy triggered by serum starvation is required for CP110 degradation at mother centrioles and ciliogenesis.

Accumulating evidence indicates that there exists a crosstalk between autophagy and ciliogenesis.^26,27^ Pampliega et al. found that knockout of *Atg5* gene promoted cilia formation under basal conditions and serum starvation in MEF cells.^26^ On the contrary, Tang et al. observed that ciliogenesis was inhibited in *Atg5*^–/–^ MEF cells upon serum deprivation,^27^ which is consistent with our data. Here, we further show that either *Atg7* knockout or autophagy inhibitors significantly suppress ciliogenesis in MEF cells under serum deprivation. These contradictory results regarding ciliogenesis in autophagy-deficient MEF cells may be due to the differences in cell confluence, which is an important factor that regulates ciliogenesis.^51^ Future studies are needed to decipher the influence of the physiological context on the interplay between autophagy and ciliogenesis.

Selective autophagy is a fundamental process mediated by autophagy receptors to specifically remove certain cargos, such as organelles and proteins.^52–54^ In general, autophagy receptors recognize specific substrates and interact with LC3 via their LIR motifs for incorporation into autophagosomes.^52,54^ The autophagosomes are further elongated and fused with lysosomes to form autolysosomes where the substrates are degraded.^52–54^ However, the role of selective autophagy in ciliogenesis remains unknown. In this report, we find that NudCL2 is a previously undescribed autophagy receptor mediating CP110 degradation at mother centrioles to initiate ciliogenesis, suggesting that selective autophagy is indispensable for ciliogenesis.

It has been reported that there exists a distal appendage protein assembly pathway in which CEP83 is at the root, branching out through CEP89 and SCLT1.^23,36^ Here, our data show that CEP83 and CEP89 not only interact with NudCL2, but are also required for the mother centriole localization of NudCL2 (Supplementary information, Fig. S8). Additionally, TTBK2 is a mother centriole-localized kinase that phosphorylates a number of distal appendage proteins to regulate ciliogenesis.^40^ In our study, we also find that TTBK2 binds to NudCL2 and recruits NudCL2 to mother centrioles (Supplementary information, Fig. S8). Further experiments are clearly required to explore the molecular mechanism by which TTBK2 regulates the mother centriole localization of NudCL2.

NudCL2 was originally characterized as a member of the mammalian nuclear distribution gene C (NudC) family, which includes NudC, NudC-like protein, and so on.^41–44,55,56^ NudC family has been documented to play critical roles in the regulation of protein stability mainly by acting as an Hsp90 co-chaperone.^44,55,56^ NudCL2 stabilizes cohesin complex subunits, E3 ligase HECT motif and RCC1-like domain-containing protein 2 (HERC2), LIS1, and myosin-9 to regulate sister chromatid cohesion, centriole amplification and cell migration, respectively.^42,43,57,58^ In this manuscript, we further characterize NudCL2 as a selective autophagy receptor governing ciliogenesis. It will be very interesting to study the functional interactions between autophagy and protein chaperoning in the future.

## Materials and Methods

### Plasmids and oligonucleotides

Full-length mouse *NudCL2* was amplified from total cDNA by RT-PCR and cloned into pGEX5X-1, pET-28a, pGFP-C1, pRFP1, pLVX-Puro, and pCS2+ vectors with an N-terminal Flag tag. Flag-M1, Flag-M2, Flag-M3, Flag-M4, His-M2, and pLVX-M2 were constructed by the mutagenesis of Flag-NudCL2, His-NudCL2, and pLVX-NudCL2 plasmids using the Mutagenesis Kit (Vazyme biotech), respectively. Fragments of NudCL2 or CP110 were amplified from their constructed plasmids by RT-PCR and cloned into pGFP-C1 vector. GST-LC3 plasmid was a kind gift from Dr. Wei Liu (Zhejiang University). Full-length mouse *CP110* cloned by RT-PCR was inserted into pFastBac-HT A (His-tag vector, Invitrogen). Oligos corresponding to the following sequences were synthesized by GenePharma: 5′-GCGGACCAGAUUUCUCAAATT-3′ for *NudCL2* RNAi-1, 5′-GGACUUUGGAGGACAGAAATT-3′ for *NudCL2* RNAi-2 and 5′-GCAATAACGTCACTGTTGA-3′ for *CP110* RNAi.

### Antibodies

Anti-NudCL2 antibody was generated as described previously.^42^ The following antibodies were commercially acquired for western blot analysis: anti-CP110 (rabbit, 1:1000, Proteintech, 12780-1-AP), anti-p62 (rabbit, 1:1000, Proteintech, 18420-1-AP), anti-β-actin (mouse, 1:2000, Proteintech, 66009-1-Ig), anti-His (mouse, 1:1000, Proteintech, 66005-1-Ig), anti-Ubiquitin (rabbit, 1:1000, Proteintech, 10201-2-AP), anti-CEP97 (rabbit, 1:1000, Proteintech, 22050-1-AP), anti-CEP83 (rabbit, 1:1000, Proteintech, 26013-1-AP), anti-CEP89 (rabbit, 1:1000, Proteintech, 24002-1-AP), anti-TTBK2 (rabbit, 1:1000, Proteintech, 15072-1-AP), anti-Rab8a (rabbit, 1:1000, Proteintech, 55296-1-AP), anti-TTBK2 (rabbit, 1:1000, Proteintech, 15072-1-AP), anti-GFP (mouse, 1:1000, Santa Cruz Biotechnology, sc-9996), anti-GST (mouse, 1:1000, Santa Cruz Biotechnology, sc-138), anti-Flag (mouse, 1:1000, Sigma-Aldrich, F3165), anti-α-tubulin (mouse, 1:1000, Sigma-Aldrich, T9026), anti-LC3B (rabbit, 1:1000, anti-Sigma-Aldrich, L7543), anti-ATG5 (rabbit, 1:500, Diagbio, db2585), anti-ATG7 (rabbit, 1:1000, Diagbio, db9934), and IgG (rabbit, Cell Signal Technology). Goat anti-mouse or anti-rabbit secondary antibodies (1:5000, LI-COR) conjugated with either Alexa Fluor 680 or IRDye 800 were used for western blot detection.

The following antibodies were used for immunofluorescence analyses: anti-acetylated-α-tubulin (mouse, 1:500, Sigma-Aldrich, T7451), anti-γ-tubulin (mouse, 1:2000, Sigma-Aldrich, T6557), anti-LC3B (rabbit, 1:200, Sigma-Aldrich, L7543), anti-LC3 (mouse, 1:200, Proteintech, 66139-1-lg), anti-centrin 2 (rabbit, 1:200, Proteintech, 15877-1-AP), anti-CEP83 (rabbit, 1:200, Proteintech, 26013-1-AP), anti-CEP89 (rabbit, 1:200, Proteintech, 24002-1-AP), anti-CEP164 (rabbit, 1:200, Proteintech, 22227-1-AP), anti-Rab8a (rabbit, 1:100, Proteintech, 55296-1-AP), anti-TTBK2 (rabbit, 1:200, Proteintech, 15072-1-AP), anti-centrin (mouse, 1:1000, Millipore, 20H5), anti-CEP164 (mouse, 1:200, Santa Cruz Biotechnology, sc-515403). The secondary antibodies for immunofluorescence analyses were Alexa Fluor 488-, 568-, and 647-conjugated anti-rabbit or anti-mouse IgGs (1:500, Invitrogen).

### Cell culture, transfection and lentiviral infection

MEF cells and HEK-293 cells were maintained in DMEM (Corning) supplemented with 10% FBS (fetal bovine serum, Gibco) and antibiotics at 37 °C in 5% CO_2_. *Atg5* and *Atg7* knockout MEFs were provided by Dr. Wei Liu (Zhejiang University). To examine cellular ciliogenesis, cells with 90% confluent were incubated in serum-free medium for 24 h. The drugs used were CQ (20 μM, Sigma-Aldrich), 3-MA (5 mM, Sigma-Aldrich) and MG132 (1 μM, MedChemExpress). Lipofectamine 2000 reagent (Invitrogen) was used for transient transfection according to the manufacturer′s instructions. Stable ectopic expression of NudCL2 and NudCL2-M2 were carried out by lentiviral infection in MEF cells. Positive cells were selected by puromycin treatment after infection for 48 h, and then plated onto 96-well plates at one cell/well for single-cell colony selection.

### Generation of *NudCL2* knockout cells by CRISPR/Cas9 system

To construct *NudCL2* knockout cells by CRISPR/Cas9-mediated genome editing, a pair of targeting sequences (5′-GCTGGCACAGAAGACCCGGCGGG-3′) were cloned into a modified one-vector system pEP330X. This plasmid was transfected into MEF cells for 48 h, and then the cells were seeded into 96-well plates for single-cell colony selection after 24 h treatment of 1 μg/mL puromycin. Following amplification of these colonies, individual genomic DNA was extracted for PCR (primers: 5′-GAGCCGTGTGCCTGCGTG-3′ and 5′-CAGTCATCCCTCTGACACCGTG-3′) and sequencing analyses. The colonies with genome editing were further verified by western blotting with anti-NudCL2 antibodies.

### Western blotting

Cell extracts were generated in TBSN buffer (20 mM Tris, pH 8.0, 150 mM NaCl, 0.5% NP-40, 5 mM EGTA, 1.5 mM EDTA, 0.5 mM Na_3_VO_4_, 20 mM p-nitrophenyl phosphate) supplemented with protease inhibitors (Roche). Then the protein samples were separated by SDS-PAGE gel, transferred to polyvinylidene fluoride membrane (Millipore), incubated with the indicated antibodies and detected by ChemiDoc Touch Imaging System (Bio-Rad) or LI-COR Odyssey imaging systems (LI-COR).

### Immunofluorescence analysis

Immunofluorescence assays were carried out as described previously.^42^ Cells grown on coverslips were fixed with cold methanol for 15 min at –20 °C, and then blocked with 3% BSA in PBS (phosphate buffered saline) for 30 min. Cells were incubated with indicated antibodies for 2 h at room temperature, followed by species-specific Alexa Fluor 488-, 555-, and 647-conjugated secondary antibodies (1:1,000, Invitrogen) for 1 h. DNA was stained with DAPI (Sigma-Aldrich). Slides were further analyzed using a laser scanning confocal microscope (Zeiss LSM880 microscopy).

### GST pull-down assays

GST pull-down assays were performed as described previously.^43^ In brief, GST, GST-NudCL2, GST-LC3, His-NudCL2, and His-NudCL2-M2 proteins were purified from *Escherichia coli* BL21. His-CP110 was purified from insect Sf9 cells using the Bac-to-Bac baculovirus expression system (Invitrogen). The purified proteins were incubated in PBS at 4 °C for 4 h, and then glutathione-agarose beads (GST pull-down) or Ni-NTA-agarose beads (His pull-down) were added for 2 h. The beads were washed and then subjected to western blotting.

### Co-IP

Co-IP was performed as previously described.^59^ Cell lysates were incubated with indicated antibodies at 4 °C overnight, and then Protein A/G Sepharose beads (Santa Cruz) were added for 4 h. For co-precipitation of Flag-tagged proteins, cells transfected with the indicated plasmids were subjected to co-IP with anti-FLAG M2 affinity gel (Sigma-Aldrich) at 4 °C for 4 h. The immunoprecipitates were washed and then processed for western blotting.

### FRET assays

The acceptor photobleaching module of Zeiss LSM880 software was employed for FRET analysis. The cells were co-stained with anti-CP110 and anti-LC3 antibodies followed by Alexa Fluor 488-conjugated anti-rabbit and 555-conjugated anti-mouse secondary antibodies, respectively. The 488-nm laser line was used to excite endogenous CP110 and the 561-nm laser line was used to excite and bleach endogenous LC3. A region containing centrosomes co-stained with CP110 and LC3 was selected for bleaching. The LC3 dots were bleached using 50 iterations of 561 nm laser line at 60% intensity. Images were taken each 6-s intervals for 5 cycles. FRET efficiency (E) was calculated as follows: E = (Dpost − Dpre)/Dpost for all Dpost > Dpre. Dpre and Dpost are donor fluorescence intensity before and after photobleaching, respectively. The fluorescence intensities were collected from the Mean ROI module of Zeiss LSM880.

### LC-MS/MS analysis

Liquid chromatography/tandem mass spectrometry (LC-MS/MS) analysis was performed as described previously.^55^ In brief, the proteins co-immunoprecipitated with anti-CP110 or anti-LC3 antibodies in MEF cells were subjected to trypsin digestion. Then, the recovered peptide mixtures were separated by reversed-phase HPLC followed by tandem mass analysis at the Research Center for Proteome Analysis, Shanghai Institutes of Biological Sciences. The peak lists of all acquired MS/MS spectra were generated by BioWorks software and then automatically searched against the human International Protein Index protein sequence database (version 3.36) using the SEQUEST algorithm.^60^

### Quantitative real-time PCR

Quantitative RT-PCR analysis was performed using a Bio-Rad CFX-Touch System with HiScript Q RT SuperMix (Vazyme, Nanjing, China). All of the reactions were performed in triplicate. GAPDH served as an internal control.

### Zebrafish maintenance

Wild-type zebrafish (strain Tübingen) was maintained at 28.5 °C using standard protocols. Zebrafish embryos were staged as described.^61^ All zebrafish experiments were performed according to the requirements by Regulation for the Use of Experimental Animals in Zhejiang Province (ETHICS CODE Permit NO. ZJU2011-1-11-009Y).

### Cloning of zebrafish *NudCL2* and *CP110*

Total RNA was extracted from zebrafish embryos with Trizol reagent (Thermo Fisher Scientific). To synthesize cDNA from the total RNA, reverse transcription was performed with Hiscript III Rverse Transcript (Vazyme) according to the manufacturer′s instructions. Zebrafish homologs of NudCL2 and CP110 were searched by Blast algorithm (U.S. National Center for Biotechnology Informaion (NCBI); http://www.ncbi.nlm.nih.gov) with the amino acid sequences of human NudCL2 and CP110. Zebrafish *NudCL2* (NM_001003539.1) and *CP110* (XM_021479933.1) were cloned by RT-PCR with total RNA extracted from zebrafish.

### Morpholinos and mRNAs

*NudCL2* MO (5′-TCTCCTCAAAATGCACCGACATATC-3′) and *CP110* MO (5′- ACTCTCCATAACTTCATTACTCAGA-3′) were synthesized by Gene Tools to target the translation of *NudCL2* and *CP110* mRNAs, respectively. Synthetic capped mRNAs were transcribed in vitro using pCS2-NudCL2, -M2, or -CP110 plasmids with MessageMachine (Ambion). 2.5 ng *NudCL2* MO was injected into zebrafish embryos at 1- to 2-cell stages. For rescue experiments, 25 pg *NudCL2* mRNA, 25 pg *M2* mRNA and 2.0 ng *CP110* MO were co-injected with *NudCL2* MO at 1- to 2-cell stages, respectively.

### Whole-mount immunostaining

The staining of cilia in zebrafish KVs was performed as previously described.^62^ Embryos at the six-somite stage were fixed in 4% paraformaldehyde overnight at 4 °C. The fixed embryos were washed with 0.1% PBST (Triton-X 100), boiled in 1 mM EDTA for 5 min and blocked with 5% bovine serum albumin in 0.1% PBST. Then, the embryos were incubated with anti-acetylated-α-tubulin (1:500) in blocking solution overnight at 4 °C. After washing in 0.1% PBST, the embryos were incubated with the Alexa Fluor 488-conjugated anti-mouse secondary antibody overnight at 4 °C. Finally, the embryos mounted in 1% low melting agarose were imaged using a 40× water immersion objective (Olympus BX61W1-FV1000).

### Whole-mount in situ hybridization

Whole-mount in situ hybridization was performed as described previously.^63^ Briefly, digoxigenin-labeled RNA probes (*cmlc2* and *foxa3*) were synthesized by in vitro transcription using T7 or SP6 RNA polymerase. Zebrafish embryos were fixed with 4% paraformaldehyde at 4 °C overnight, and then hybridized with *cmlc2* or *foxa3* probe at 68 °C overnight. Alkaline phosphatase-coupled antidigoxigenin antibody (1:3000 dilution, Roche) was used to detect digoxigenin probes, and NBT/BCIP solution (BM purple, Roche) was used as the chromogenic substrate to visualize RNA signal.

### Statistics

Data are representative of at least three independent experiments. Means and standard deviations (SD) were calculated and are shown in the graphs. Student’s *t*-test was used to determine statistically significant differences between two groups.

## Supplementary information

Supplementary information, Fig. S1

Supplementary information, Fig. S2

Supplementary information, Fig. S3

Supplementary information, Fig. S4

Supplementary information, Fig. S5

Supplementary information, Fig. S6

Supplementary information, Fig. S7

Supplementary information, Fig. S8

Supplementary information, Fig. S9

Supplementary information, Fig. S10

Supplementary information, Fig. S11

Supplementary information, Fig. S12

Supplementary information, Fig. S13
