# Supplementary material for: NudCL2 is an autophagy receptor that mediates selective autophagic degradation of CP110 at mother centrioles to promote ciliogenesis
Source: Cell Res. 2021 Sep 3;31(11):1199–211. doi: 10.1038/s41422-021-00560-3 (PMC8563757; doi:10.1038/s41422-021-00560-3)
Supplement: Supplementary file 1 — Supplementary information, Fig. S1 [file 41422_2021_560_MOESM1_ESM.pdf]

Supplementary information, Figure S1

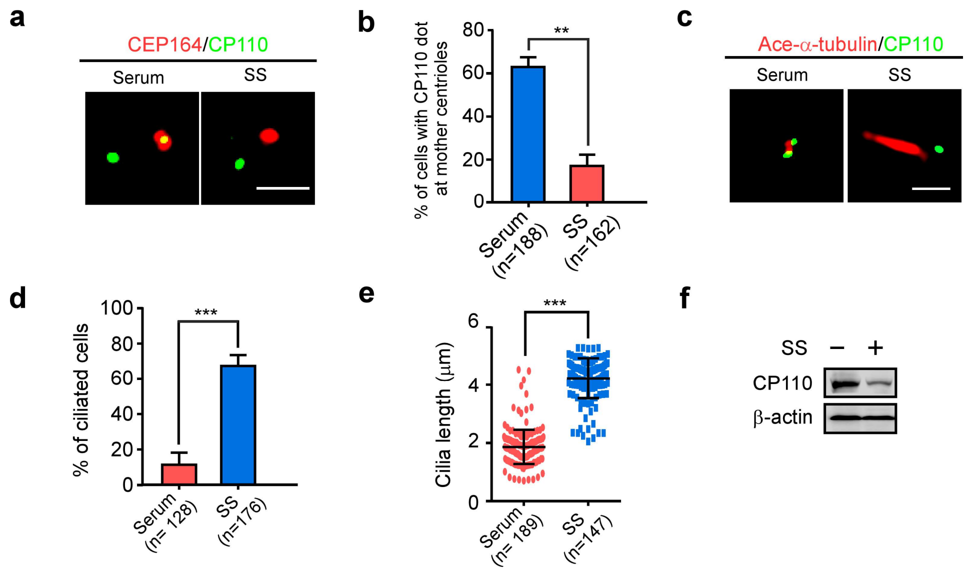

Supplementary information, Fig. S1 Serum starvation promotes the removal of mother centriole-localized CP110 and ciliogenesis. MEF cells were cultured with or without serum for 24 h, and subjected to the following analyses. **a, b** Immunofluorescence analysis of CP110 and CEP164 (a mother centriole marker). SS, serum starvation. Scale bar, 2 μm. The percentage of cells with CP110 localized at mother centrioles was calculated. **c-e** Immunostaining with anti-CP110 and anti-ace-α-tubulin (acetylated-α-tubulin) antibodies. Scale bar, 2 μm. The cells with cilia were calculated. Cilia length was measured using ImageJ software. **f** Western analysis of CP110 in MEF cells with or without serum. β-actin was used as a loading control. Quantitative data are expressed as the mean ± SD (at least three independent experiments). n, sample size. **$P < 0.01$ and ***$P < 0.001$, Student's *t*-test.
