# Supplementary material for: NudCL2 is an autophagy receptor that mediates selective autophagic degradation of CP110 at mother centrioles to promote ciliogenesis
Source: Cell Res. 2021 Sep 3;31(11):1199–211. doi: 10.1038/s41422-021-00560-3 (PMC8563757; doi:10.1038/s41422-021-00560-3)
Supplement: Supplementary file 2 — Supplementary information, Fig. S2 [file 41422_2021_560_MOESM2_ESM.pdf]

Supplementary information, Figure S2

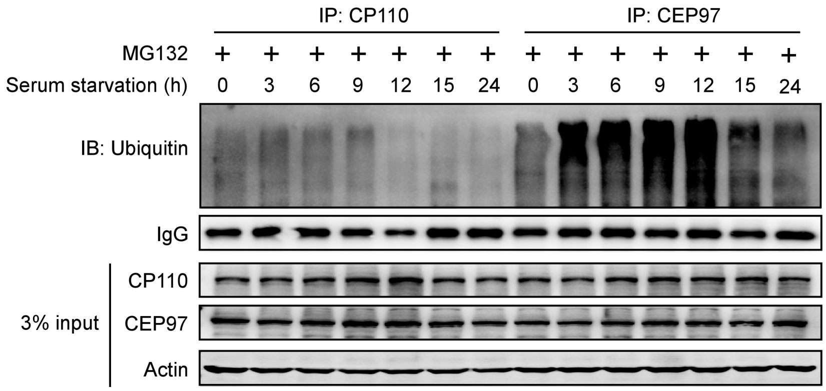

Supplementary information, Fig. S2 The ubiqutination level of CP110 is not obviously changed during serum starvation. MEF cells treated with 1 μM MG132 were subjected to serum deprivation at the indicated time points. The cells were then lysed and applied for co-immunoprecipitation with the indicated antibodies.
