# Supplementary material for: NudCL2 is an autophagy receptor that mediates selective autophagic degradation of CP110 at mother centrioles to promote ciliogenesis
Source: Cell Res. 2021 Sep 3;31(11):1199–211. doi: 10.1038/s41422-021-00560-3 (PMC8563757; doi:10.1038/s41422-021-00560-3)
Supplement: Supplementary file 3 — Supplementary information, Fig. S3 [file 41422_2021_560_MOESM3_ESM.pdf]

Supplementary information, Figure S3

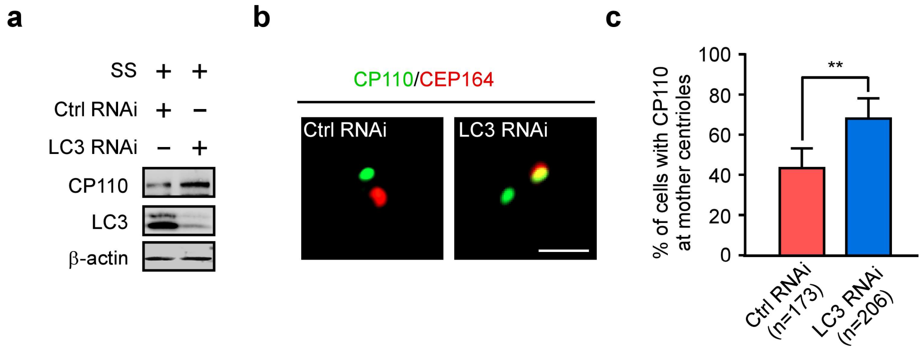

Supplementary information, Fig. S3 Knockdown of LC3 increases CP110 levels and impairs CP110 removal from mother centrioles. MEF cells were transfected with the indicated siRNAs, cultured without serum for 24 h, and processed for the following analyses. **a** Western blotting of the indicated proteins. β-actin, a loading control. **b** Immunofluorescence analysis was carried out by using anti-CEP164 and anti-CP110 antibodies. Scale bar, 1 µm. **c** The percentage of cells with CP110 at mother centrioles was counted. Quantitative data are expressed as the mean ± SD (three independent experiments). n, sample size. $^{**}P < 0.01$, Student's *t*-test.
