# Supplementary material for: NudCL2 is an autophagy receptor that mediates selective autophagic degradation of CP110 at mother centrioles to promote ciliogenesis
Source: Cell Res. 2021 Sep 3;31(11):1199–211. doi: 10.1038/s41422-021-00560-3 (PMC8563757; doi:10.1038/s41422-021-00560-3)
Supplement: Supplementary file 4 — Supplementary information, Fig. S4 [file 41422_2021_560_MOESM4_ESM.pdf]

Supplementary information, Figure S4

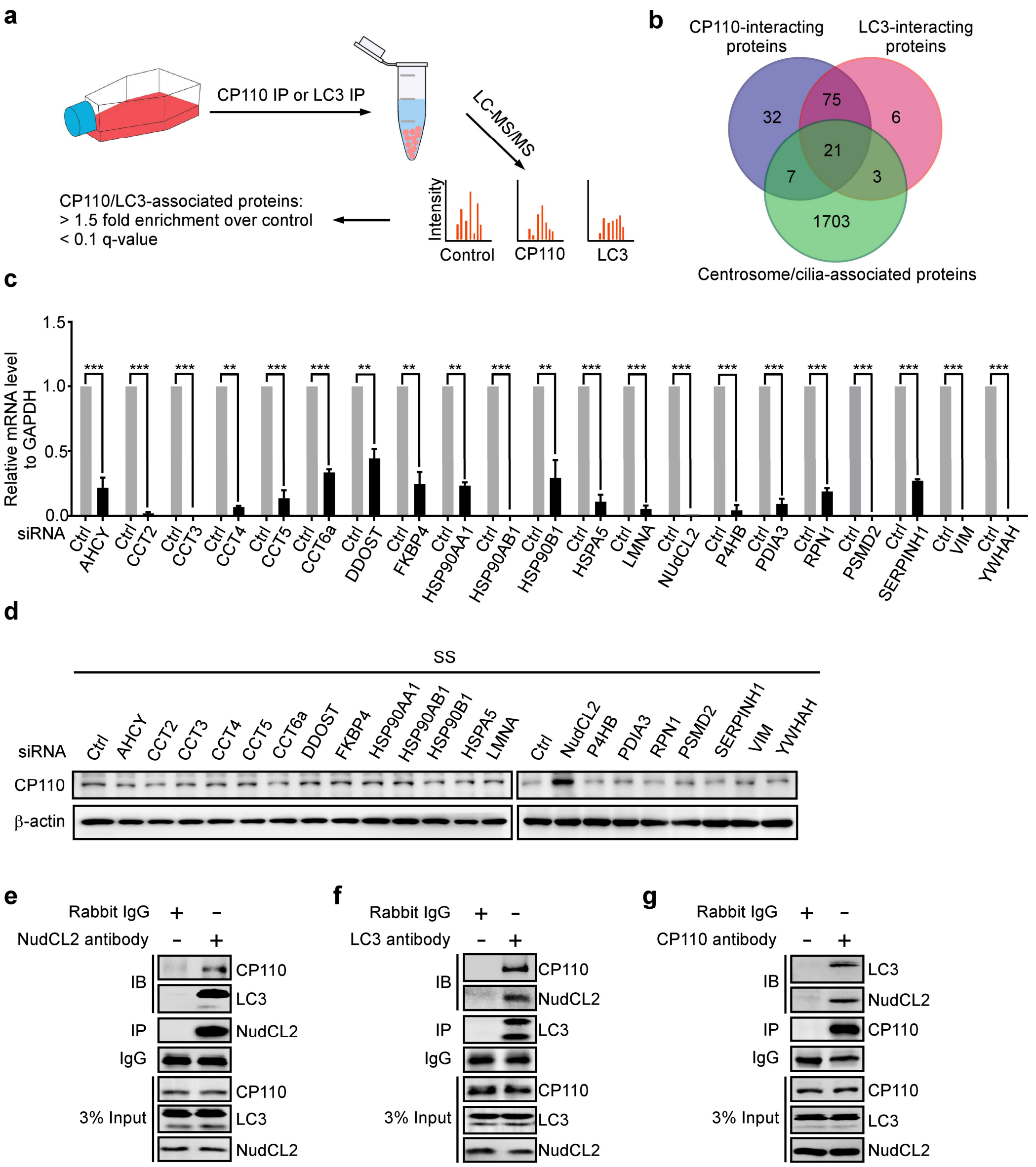

Supplementary information, Fig. S4 NudCL2 is associated with LC3 and CP110. **a** Schematic of the screening of LC3- or CP110-interacting proteins in MEF cells by using co-immunoprecipitation in combination with mass spectrometry. **b** Venn diagram of the overlapping proteins in the indicated groups. **c** MEF cells transfected with the indicated siRNAs were subjected to quantitative real-time PCR (polymerase chain reaction) analysis. Adenosylhomocysteinase (AHCY); Chaperonin containing TCP1 subunit 2 (CCT2); Chaperonin containing TCP1 subunit 3 (CCT3); Chaperonin

containing TCP1 subunit 4 (CCT4); Chaperonin containing TCP1 subunit 5 (CCT5); Chaperonin containing TCP1 subunit 6A (CCT6a); Dolichyl-diphosphooligosaccharide--protein glycosyltransferase non-catalytic subunit (DDOST); FKBP prolyl isomerase 4 (FKBP4); Heat shock protein 90 alpha family class A member 1 (HSP90AA1); Heat shock protein HSP90-beta (HSPAB1); Heat shock protein 90 beta family member 1 (HSP90B1); Heat shock protein A5 (HSPA5); Lamin A/C (LMNA); NudC-like protein 2 (NudCL2); Prolyl 4-hydroxylase beta chain (P4HB); Protein disulfide isomerase family A member 3 (PDIA3); Ribophorin I (RPN1); Proteasome 26S subunit (PSMD2); Serpin family H member 1 (SERPINH1); Vimentin (VIM); Tyrosine 3-monooxygenase/tryptophan 5-monooxygenase activation protein η (YWHAH). *GAPDH* acts as an internal control. **d** MEF cells were transfected with the indicated siRNAs, treated with serum starvation for 24 h, and then processed for western blotting with anti-CP110 antibody. β-actin, a loading control. **e-g** Total lysates from MEF cells were subjected to co-immunoprecipitation analyses with anti-NudCL2, anti-LC3 and anti-CP110 antibodies. 3% of input is shown. Quantitative data are expressed as the mean ± SD (at least three independent experiments). $^{**}P < 0.01$ and $^{***}P < 0.001$, Student's *t*-test.
