# Supplementary material for: NudCL2 is an autophagy receptor that mediates selective autophagic degradation of CP110 at mother centrioles to promote ciliogenesis
Source: Cell Res. 2021 Sep 3;31(11):1199–211. doi: 10.1038/s41422-021-00560-3 (PMC8563757; doi:10.1038/s41422-021-00560-3)
Supplement: Supplementary file 5 — Supplementary information, Fig. S5 [file 41422_2021_560_MOESM5_ESM.pdf]

Supplementary information, Figure S5

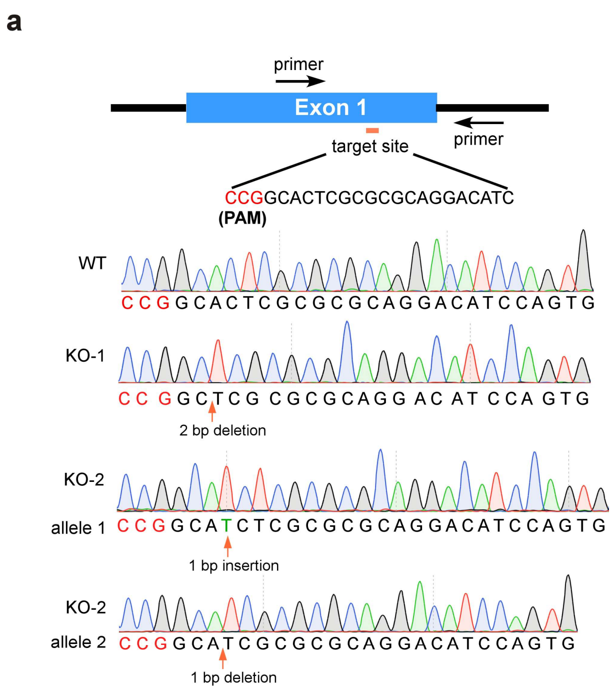

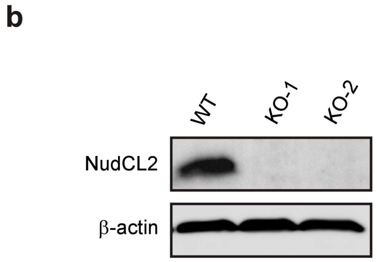

Supplementary information, Fig. S5 Disruption of *NudCL2* gene in MEF cells. a Diagram of the sgRNA target site (underlined) and the sequence of indels in the *NudCL2* locus in MEF cells generated by the CRISPR/Cas9 system. The PAM (protospacer adjacent motif) site is indicated in red. **b** TA cloning of PCR products from genomic DNA extracted from wild-type (WT) and *NudCL2* knockout MEF cells (KO-1 and KO-2). The orange arrows indicate base mutation (deletion or insertion) in the knockout cells. **c** Immunoblotting of endogenous NudCL2 in WT and *NudCL2* knockout MEF cells. β-actin was used as a loading control.
