# Supplementary material for: NudCL2 is an autophagy receptor that mediates selective autophagic degradation of CP110 at mother centrioles to promote ciliogenesis
Source: Cell Res. 2021 Sep 3;31(11):1199–211. doi: 10.1038/s41422-021-00560-3 (PMC8563757; doi:10.1038/s41422-021-00560-3)
Supplement: Supplementary file 6 — Supplementary information, Fig. S6 [file 41422_2021_560_MOESM6_ESM.pdf]

Supplementary information, Figure S6

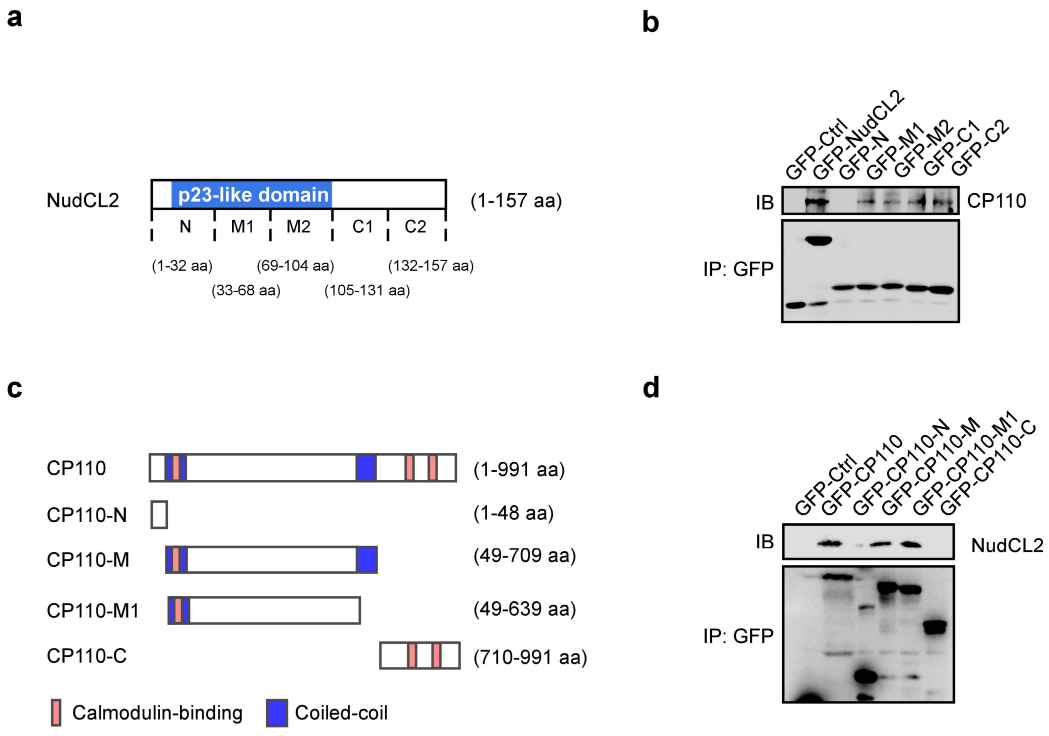

Supplementary information, Fig. S6 Mapping of the NudCL2-CP110 interacting regions. a Schematic of NudCL2 truncation mutants. **b** The NudCL2 truncation mutants fused with GFP were transfected into 293 cells. The cell lysates were immunoprecipitated by anti-GFP antibody and subjected to western blotting. **c** Summary of CP110 truncation mutants. **d** The CP110 mutants were fused with GFP and transfected into 293 cells. The cells were lysed, immunoprecipitated by anti-GFP antibody, and processed for western analysis.
