# Supplementary material for: NudCL2 is an autophagy receptor that mediates selective autophagic degradation of CP110 at mother centrioles to promote ciliogenesis
Source: Cell Res. 2021 Sep 3;31(11):1199–211. doi: 10.1038/s41422-021-00560-3 (PMC8563757; doi:10.1038/s41422-021-00560-3)
Supplement: Supplementary file 7 — Supplementary information, Fig. S7 [file 41422_2021_560_MOESM7_ESM.pdf]

Supplementary information, Figure S7

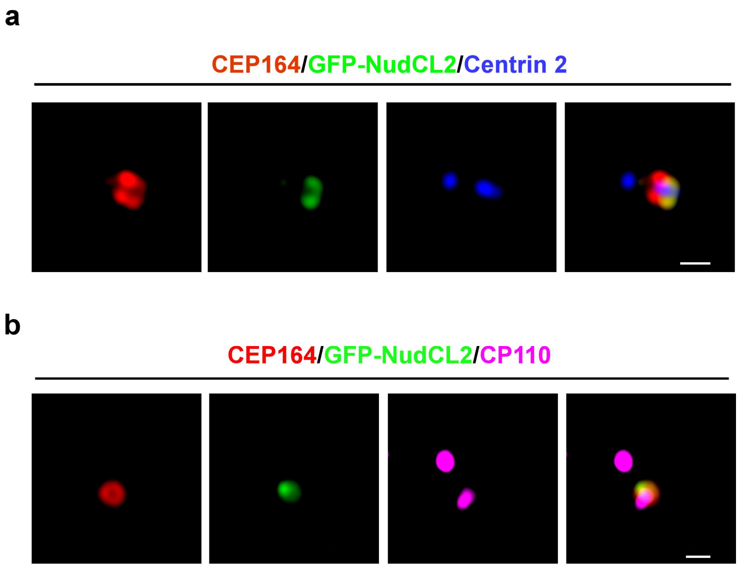

Supplementary information, Fig. S7 The localization of NudCL2 at mother centrioles. MEF cells transfected with GFP-NudCL2 were applied for immunostaining with anti-CEP164 and anti-centrin 2 or anti-CP110 antibodies and subjected to Airyscan confocal super-resolution microscopy. **a** Representative super-resolution images of CEP164 and NudCL2 co-stained with centrin 2. **b** Representative images of CEP164 and GFP-NudCL2 co-stained with CP110. Scale bars, 0.5 μm.
