# Supplementary material for: NudCL2 is an autophagy receptor that mediates selective autophagic degradation of CP110 at mother centrioles to promote ciliogenesis
Source: Cell Res. 2021 Sep 3;31(11):1199–211. doi: 10.1038/s41422-021-00560-3 (PMC8563757; doi:10.1038/s41422-021-00560-3)
Supplement: Supplementary file 8 — Supplementary information, Fig. S8 [file 41422_2021_560_MOESM8_ESM.pdf]

Supplementary information, Figure S8

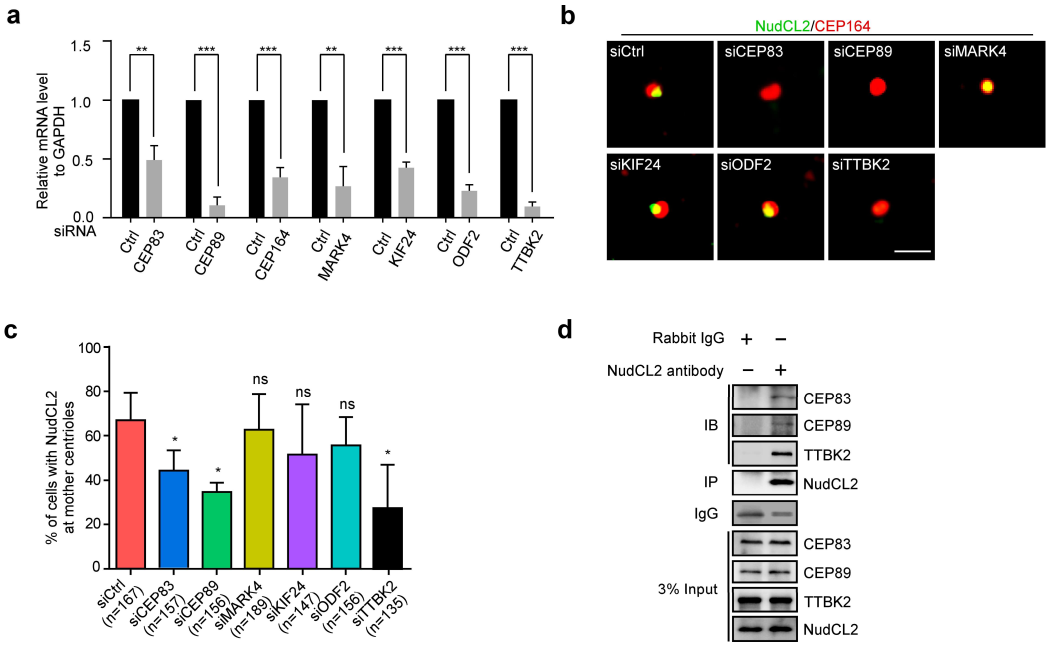

Supplementary information, Fig. S8 Depletion of mother centriole-localized proteins influences the localization of NudCL2 at mother centriole. MEF cells transfected with the indicated siRNAs were subjected to the following experiments. **a** Quantitative real-time PCR analysis of the indicated mRNA expression. *GAPDH* acts as an internal control. Centrosomal protein 83 (CEP83); Centrosomal protein 89 (CEP89); Centrosomal protein 164 (CEP164); Microtubule affinity regulating kinase 4 (MARK4); Kinesin family member 24 (KIF24); Outer dense fiber of sperm tails 2 (ODF2); Tau tubulin kinase 2 (TTBK2). **b, c** Co-localization of NudCL2 with CEP164 in cells transfected with the indicated siRNAs. The percentage of cells with mother centriole-localized NudCL2 was calculated. **d** Total lysates from MEF cells were subjected to co-immunoprecipitation analyses with anti-NudCL2 antibody. 3% of input is shown. Quantitative data are expressed as the mean ± SD (at least three independent experiments). Scale bars, 2 μm. n, sample size. **P* < 0.05, ***P* < 0.01, ****P* < 0.001, ns, not significant (*P* > 0.05), Student's *t*-test.
