# Supplementary material for: NudCL2 is an autophagy receptor that mediates selective autophagic degradation of CP110 at mother centrioles to promote ciliogenesis
Source: Cell Res. 2021 Sep 3;31(11):1199–211. doi: 10.1038/s41422-021-00560-3 (PMC8563757; doi:10.1038/s41422-021-00560-3)
Supplement: Supplementary file 9 — Supplementary information, Fig. S9 [file 41422_2021_560_MOESM9_ESM.pdf]

Supplementary information, Figure S9

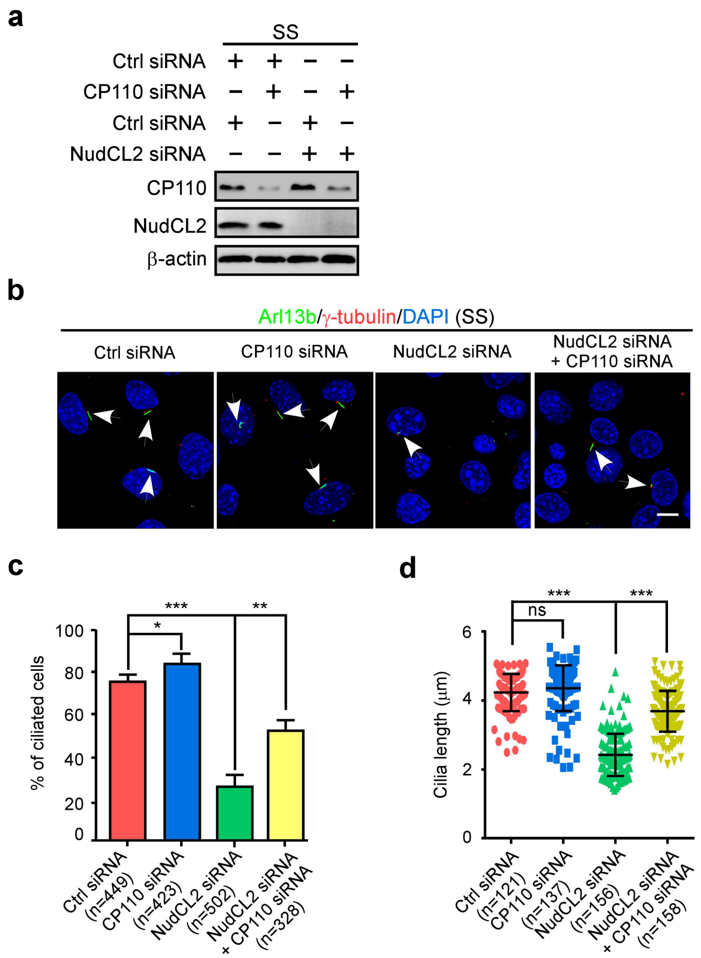

Supplementary information, Fig. S9 NudCL2 regulates CP110 degradation to promote ciliogenesis. MEF cells transfected with the indicated siRNAs were starved for 24 h, and processed for the following analyses. **a** Western blotting of NudCL2 and CP110. β-actin, a loading control. **b-d** Immunofluorescence analysis with anti-Arl13b and anti-γ-tubulin antibodies. Scale bar, 10 μm. The cells with cilia were calculated. Cilia length was also measured using ImageJ software. Cilia are indicated by the white arrows. Quantitative data from at least three independent experiments are shown as the mean ± SD. n, sample size. *$P < 0.05$, **$P < 0.01$, ***$P < 0.001$, ns, not significant ($P > 0.05$), Student's *t*-test.
