# Supplementary material for: NudCL2 is an autophagy receptor that mediates selective autophagic degradation of CP110 at mother centrioles to promote ciliogenesis
Source: Cell Res. 2021 Sep 3;31(11):1199–211. doi: 10.1038/s41422-021-00560-3 (PMC8563757; doi:10.1038/s41422-021-00560-3)
Supplement: Supplementary file 10 — Supplementary information, Fig. S10 [file 41422_2021_560_MOESM10_ESM.pdf]

Supplementary information, Figure S10

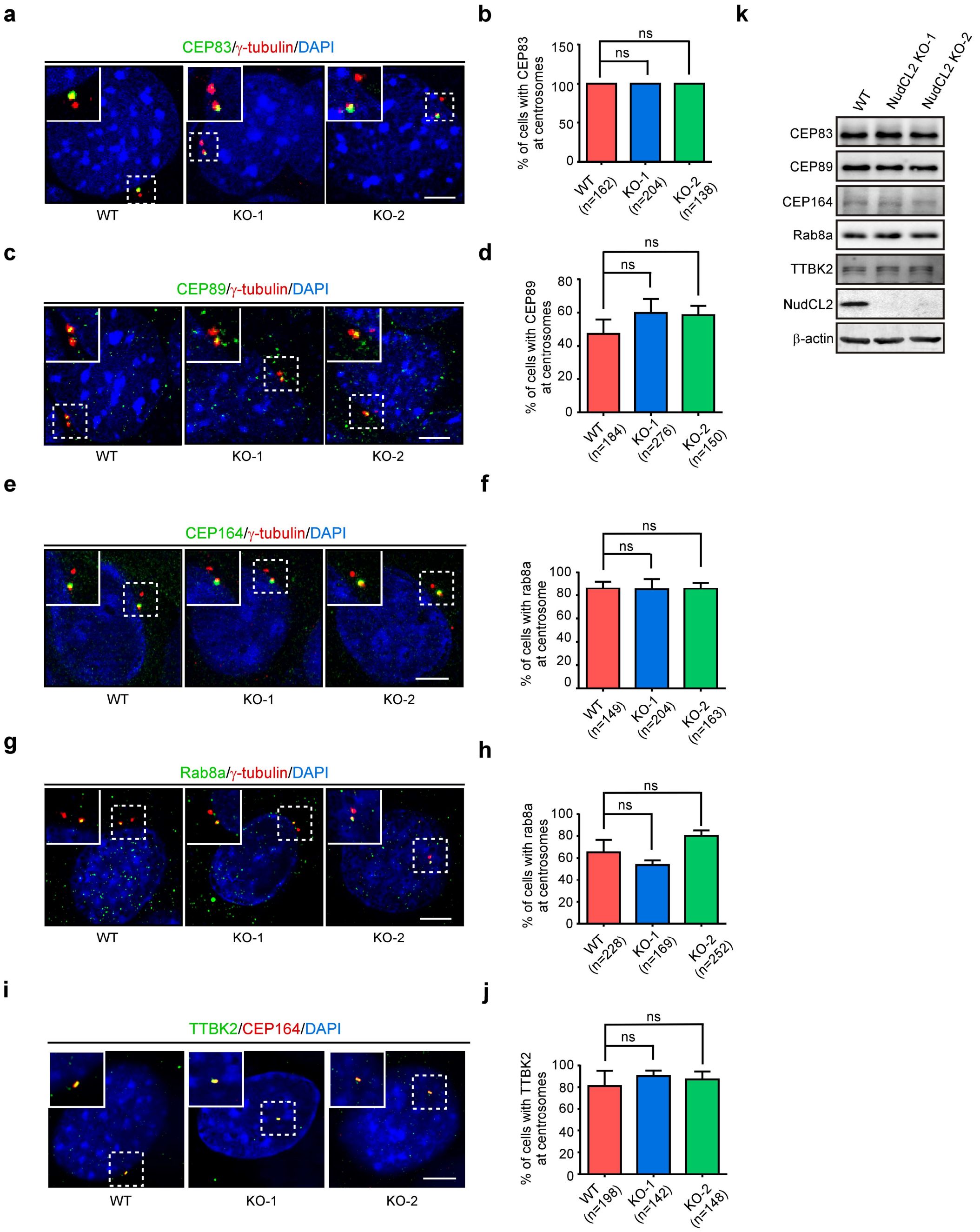

Supplementary information, Fig. S10 The effect of *NudCL2* knockout on the early steps of ciliogenesis. *NudCL2* knockout MEF cells treated with serum starvation for 24 h were subjected to the following analyses. **a**, **c**, **e**, **g**, **i** Immunostaining of the indicated proteins. High magnification images of the boxed areas are shown. **b**, **d**, **f**, **h**, **j** The percentage of cells with the indicated proteins at

centrosomes or mother centrioles was measured. Scale bars, 5 μm. Quantitative data are expressed as the mean ± SD (three independent experiments). **k** Western blotting with the indicated antibodies. β-actin, a loading control. n, sample size. ns, not significant ($P > 0.05$), Student's *t*-test.
