# Supplementary material for: NudCL2 is an autophagy receptor that mediates selective autophagic degradation of CP110 at mother centrioles to promote ciliogenesis
Source: Cell Res. 2021 Sep 3;31(11):1199–211. doi: 10.1038/s41422-021-00560-3 (PMC8563757; doi:10.1038/s41422-021-00560-3)
Supplement: Supplementary file 11 — Supplementary information, Fig. S11 [file 41422_2021_560_MOESM11_ESM.pdf]

Supplementary information, Figure S11

a

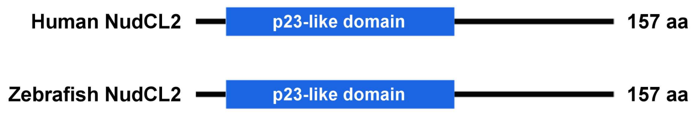

b

% Identity (similarity)

	H.sapiens	*M.musculus*	*X.tropicalis*	*D.rerio*
M.musculus	99.4% (100%)			
X.tropicalis	77.1% (89.8%)	77.1% (89.8%)		
D.rerio	70.1% (81.5%)	70.1% (81.5%)	74.5% (84.1%)	100% (100%)

Supplementary information, Fig. S11 Characterization of zebrafish *NudCL2* gene. a Schematic diagram of the conserved domain of human and zebrafish NudCL2 amino acid sequences. The conserved p23-like domain is shown in blue filled bars. **b** Comparison of NudCL2 amino acid sequences in the indicated species.
