# Supplementary material for: NudCL2 is an autophagy receptor that mediates selective autophagic degradation of CP110 at mother centrioles to promote ciliogenesis
Source: Cell Res. 2021 Sep 3;31(11):1199–211. doi: 10.1038/s41422-021-00560-3 (PMC8563757; doi:10.1038/s41422-021-00560-3)
Supplement: Supplementary file 12 — Supplementary information, Fig. S12 [file 41422_2021_560_MOESM12_ESM.pdf]

Supplementary information, Figure S12

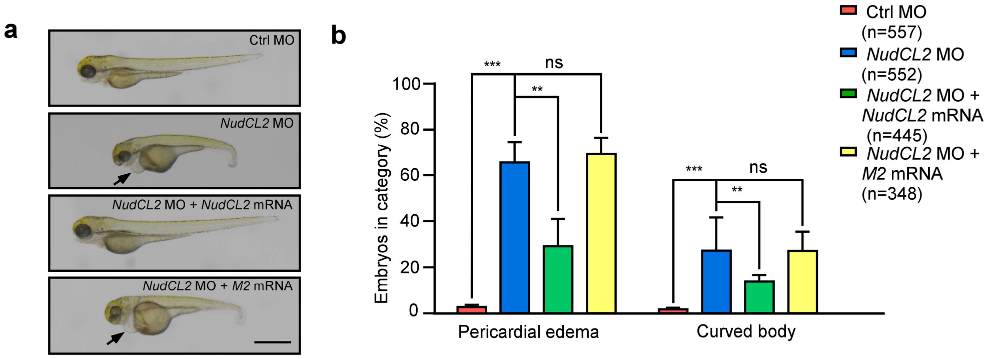

Supplementary information, Fig. S12 *NudCL2* depletion induces multiple ciliary defects in zebrafish. Embryos injected with the indicated MOs and mRNAs were collected at 72 hpf and subjected to the following analyses. **a** Bright-field micrographs of the ciliary phenotypes of *NudCL2* morphants. Scale bar, 500 μm. **b** The percentages of embryos with the indicated phenotypes. The data of at least three independent experiments are presented as mean ± SD. n, sample size. $^{**}P < 0.01$, $^{***}P < 0.001$, ns, not significant ($P > 0.05$), Student's *t*-test.
