# Supplementary material for: NudCL2 is an autophagy receptor that mediates selective autophagic degradation of CP110 at mother centrioles to promote ciliogenesis
Source: Cell Res. 2021 Sep 3;31(11):1199–211. doi: 10.1038/s41422-021-00560-3 (PMC8563757; doi:10.1038/s41422-021-00560-3)
Supplement: Supplementary file 13 — Supplementary information, Fig. S13 [file 41422_2021_560_MOESM13_ESM.pdf]

Supplementary information, Figure S13

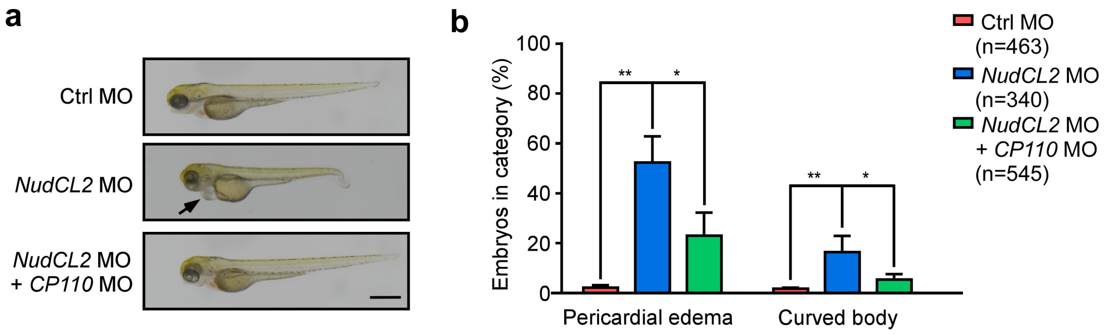

Supplementary information, Fig. S13 Knockdown of *CP110* rescues the ciliary defects in *NudCL2* morphants. Embryos injected with the indicated MOs were harvested at 72 hpf and processed for the following analyses. **a** Bright-field micrographs of the ciliary phenotypes of *NudCL2* morphants. Scale bar, 500 μm. **b** The percentages of embryos with the indicated phenotypes. The data of at least three independent experiments are presented as mean ± SD. n, sample size. $^*P < 0.05$, $^{**}P < 0.01$, Student's *t*-test.
